# The Molecular Mechanisms Underlying Prostaglandin D_2_-Induced Neuritogenesis in Motor Neuron-Like NSC-34 Cells

**DOI:** 10.3390/cells9040934

**Published:** 2020-04-10

**Authors:** Hiroshi Nango, Yasuhiro Kosuge, Nana Yoshimura, Hiroko Miyagishi, Takanori Kanazawa, Kaname Hashizaki, Toyofumi Suzuki, Kumiko Ishige

**Affiliations:** 1 Laboratory of Pharmacology, School of Pharmacy, Nihon University, 7-7-1 Narashinodai, Funabashi-shi, Chiba 274-8555, Japan; phhi16003@g.nihon-u.ac.jp (H.N.); phna14246@g.nihon-u.ac.jp (N.Y.); miyagishi.hiroko@nihon-u.ac.jp (H.M.); ishige.kumiko@nihon-u.ac.jp (K.I.); 2 Laboratory of Pharmaceutics, School of Pharmacy, Nihon University, 7-7-1 Narashinodai, Funabashi-shi, Chiba 274-8555, Japan; kanazawa.takanori02@nihon-u.ac.jp (T.K.); suzuki.toyofumi@nihon-u.ac.jp (T.S.); 3 Laboratory of Physical Chemistry, School of Pharmacy, Nihon University, 7-7-1 Narashinodai, Funabashi-shi, Chiba 274-8555, Japan; hashizaki.kaname@nihon-u.ac.jp

**Keywords:** prostaglandin D_2_, 15-deoxy-Δ^12,14^-prostaglandin J_2_, peroxisome proliferator-activated receptor γ, motor neurons, neurite outgrowth, NSC-34 cells

## Abstract

Prostaglandins are a group of physiologically active lipid compounds derived from arachidonic acid. Our previous study has found that prostaglandin E_2_ promotes neurite outgrowth in NSC-34 cells, which are a model for motor neuron development. However, the effects of other prostaglandins on neuronal differentiation are poorly understood. The present study investigated the effect of prostaglandin D_2_ (PGD_2_) on neuritogenesis in NSC-34 cells. Exposure to PGD_2_ resulted in increased percentages of neurite-bearing cells and neurite length. Although D-prostanoid receptor (DP) 1 and DP2 were dominantly expressed in the cells, BW245C (a DP1 agonist) and 15(R)-15-methyl PGD_2_ (a DP2 agonist) had no effect on neurite outgrowth. Enzyme-linked immunosorbent assay demonstrated that PGD_2_ was converted to 15-deoxy-Δ^12,14^-prostaglandin J_2_ (15d-PGJ_2_) under cell-free conditions. Exogenously applied 15d-PGJ_2_ mimicked the effect of PGD_2_ on neurite outgrowth. GW9662, a peroxisome proliferator-activated receptor–gamma (PPARγ) antagonist, suppressed PGD_2_-induced neurite outgrowth. Moreover, PGD_2_ and 15d-PGJ_2_ increased the protein expression of Islet-1 (the earliest marker of developing motor neurons), and these increases were suppressed by co-treatment with GW9662. These results suggest that PGD_2_ induces neuritogenesis in NSC-34 cells and that PGD_2_-induced neurite outgrowth was mediated by the activation of PPARγ through the metabolite 15d-PGJ_2_.

## 1. Introduction

Neuritogenesis is an early event in the differentiation of neural progenitor cells into neurons and enables neurons to develop axons and dendrites with which they connect to other cells and receive and transmit electrical signals [1]. Spinal motor neurons innervate muscle contraction by extending long axons that project from the ventral horn of the spinal cord gray matter directly to peripheral skeletal muscle [2]. Degeneration and loss of spinal motor neurons cause progressive and fatal motor neuron diseases such as amyotrophic lateral sclerosis (ALS), primary lateral sclerosis, and spinal muscular atrophy. More recently, induced pluripotent stem cell-derived motor neurons have been expected to elucidate the pathogenesis and facilitate the identification of specific drugs for the treatment of such neurodegenerative diseases [3]. Understanding the mechanism involved in neuritogenesis and differentiation of spinal motor neurons could pave the way for the development of regenerative therapies for motor neuron diseases. NSC-34 cells are produced by the fusion of mouse neuroblastoma cells with motor neuron-enriched mouse spinal cord cells [4]. Differentiated NSC-34 cells induced by serum deprivation and additional treatment with all-*trans* retinoic acid exhibit the unique morphological and physiological characteristics of motor neurons, including neurite outgrowth, expression of motor neuron-specific marker proteins HB9 and Islet-1, and acetylcholine synthesis and storage [5,6]. Furthermore, undifferentiated NSC-34 cells are widely used as motor neuron progenitor cells in the search for small molecular compounds that induce motor neuron differentiation [7,8,9,10].

Prostaglandins are small lipid inflammatory mediators derived from arachidonic acid by multiple enzymatic reactions and are involved in a wide array of physiological and pathophysiological responses [11]. In particular, prostaglandin E_2_ (PGE_2_) and D_2_ (PGD_2_) are the major products of prostaglandins. Arachidonic acid is liberated from cellular membranes by phospholipases A_2_ and is converted to prostaglandin H_2_ (PGH_2_) by cyclooxygenase-1 and -2. Subsequently, PGH_2_ is converted to PGE_2_ by PGE synthase [11] or PGD_2_ by PGD synthase (PGDS), respectively. [12]. Prostaglandins exert their actions by acting on a group of G-protein-coupled receptors. For example, PGE_2_ mainly binds to four subtypes of E-prostanoid receptor (EP1–4) [13]. PGE_2_ promotes neuritogenesis in dorsal root ganglion neurons via EP2 (coupled to Gs protein) [14] and in sensory neuron-like ND7/23 cells via EP4 (coupled to Gs protein) [15]. Recently, our research has demonstrated that PGE_2_ induces neurite outgrowth by activating EP2 in NSC-34 cells [16]. This suggests that PGE_2_ is involved in neuritogenesis and the differentiation of various neurons including motor neurons. However, the role of prostaglandins other than PGE_2_ on neuronal differentiation has not been investigated in neurons or their model cells. So far, two isoforms of PGDS are known, lipocalin-type and hematopoietic PGDS [17]. Lipocalin-type PGDS mRNA has been reported to be abundantly expressed in the thalamus, brainstem, cerebellum, and spinal cord [18]. Hematopoietic PGDS is expressed in microglial cells of the mouse cerebral cortex during early postnatal development [19] and in the adult rat cerebellum [20]. In the human brain, the amount of PGD_2_ is high in the pineal body, pituitary gland, olfactory bulb, and hypothalamus [21]. It is noteworthy that PGD_2_ is the most abundant eicosanoid in the mouse spinal cord [22] and is present in several regions of the central nervous system (CNS), including the spinal cord. Synthesized PGD_2_ elicits its downstream effects by activating two G-protein-coupled receptors, D-prostanoid receptor (DP) 1 and DP2. DP1 is coupled to adenylate cyclase via a Gs protein, while DP2 inhibits adenylate cyclase via Gi protein [12]. DP1 and DP2 proteins have been detected in motor neurons of adult mouse spinal cords with fluorescent immunohistochemistry [23]. Moreover, PGD_2_ are nonenzymatically metabolized to prostaglandin J_2_ (PGJ_2_), Δ^12^-PGJ_2_, and 15-deoxy-Δ^12,14^-PGJ_2_ (15d-PGJ_2_) [12]. It has been reported that 15d-PGJ_2_ acts as an agonist of the nuclear receptor peroxisome proliferator-activated receptor γ (PPARγ) [24]. Indeed, 15d-PGJ_2_ plays an important role in neurite outgrowth of rat embryonic midbrain cells in a PPARγ-dependent manner [25]. However, unlike PGE_2_, it is unknown whether PGD_2_ and/or 15d-PGJ_2_ exert neurite outgrowth in motor neurons. In this study, we sought to elucidate the effect of PGD_2_ on neurite outgrowth in motor neurons using NSC-34 cells. We found that exogenously applied PGD_2_ was converted to 15d-PGJ_2_ and subsequently induced neurite outgrowth, which was mediated by PPARγ but not by DP in motor neuron-like cells.

## 2. Materials and Methods

### 2.1. Materials

All chemicals were purchased from Wako (Osaka, Japan) unless stated otherwise. PGD_2_, 15d-PGJ_2_, BW 245C, 15(R)-15-methyl PGD_2_, MK0524, CAY10471, and GW9662 were obtained from Cayman Chemical (Ann Arbor, MI, USA). These compounds were dissolved in dimethyl sulfoxide (DMSO; Sigma–Aldrich, St. Louis, MO, USA). The primary antibodies used were against DP1 (Cayman Chemical, Ann Arbor, MI, USA, diluted 1:1000), DP2 (Novus Biologicals, Centennial CO, USA, diluted 1:1000), Islet-1 (Cell Signaling Technology, Danvers, MA, USA, diluted 1:1000), and β-actin (Sigma-Aldrich, St. Louis, MO, USA, diluted 1:1000).

### 2.2. Cell Culture

NSC-34 cells (provided by Dr. Neil Cashman, University of Toronto, ON, Canada) were cultured to a maximum of 15 passages in a medium consisting of Dulbecco’s Modified Eagle Medium (DMEM; Sigma–Aldrich, St. Louis, MO, USA) supplemented with 10% fetal bovine serum (FBS; Life Technologies Corporation, Carlsbad, CA, USA) and 1% penicillin–streptomycin (Life Technologies Corporation, Carlsbad, CA, USA). Cultures were incubated at 37 °C and 5% CO_2_ in a humidified atmosphere. For experimentation, NSC-34 cells were seeded onto Corning BioCoat™ Poly-L-Lysine TC-Treated Culture Dishes (Corning Inc., Corning, NY, USA) at a density of 12,500 cells/cm^2^ and incubated for 24 h according to the method described previously [16].

### 2.3. Western Blotting

The Western blot analysis was performed as described previously [26,27]. Briefly, NSC-34 cells were lysed in a radioimmunoprecipitation buffer containing 150 mM NaCl, 1% Nonidet P-40, 0.5% sodium deoxycholate, 0.1% SDS, 50 mM Tris-HCl (pH 8.0), 1% Triton X-100, 5 mM EDTA, phosphatase inhibitor (Sigma–Aldrich, St. Louis, MO, USA), and protease inhibitor cocktail (Roche, Basel, Switzerland). The proteins were separated by sodium dodecyl sulfate–polyacrylamide gel electrophoresis, transferred onto the membranes, and probed with the antibodies. The following primary antibodies were used: polyclonal anti-DP1 (1:1000), polyclonal anti-CRTH2/GRP44 (DP2) (1:1000), monoclonal anti-Islet-1 (1:1000), and monoclonal anti-β-actin (Sigma–Aldrich, St. Louis, MO, USA). An HRP-conjugated secondary antibody (Santa Cruz Biotechnology, Dallas, TX, USA) was used. Immunoreactive bands were detected by enhanced chemiluminescence (GE Healthcare, Buckinghamshire, UK). The optical density of the bands was quantified using Scion imaging software 4.0.3.2 (Scion, Frederick, MD, USA).

### 2.4. Neurite Outgrowth Assay

Phase-contrast micrographs were captured using an inverted microscope (IX70, Olympus, Tokyo, Japan) with i-NTER LENS (Microscope Network Co. Ltd., Saitama, Japan). Afterwards, 50 cells per condition were randomly chosen to estimate the number of neurite-bearing cells using ImageJ (National Institute of Health, Bethesda, MD, USA) [28]. The percentage of neurite-bearing cells was calculated as the ratio of cells bearing neurite processes >1 cell diameter in length according to the method described previously [16]. The neurite length on each neurite-bearing cell was measured as the distance from the soma to the end of the neurite using NeuronJ, an ImageJ plugin originally developed for neurite tracing and analysis [29].

### 2.5. Enzyme-Linked Immunosorbent Assay (ELISA)

Quantification of PGD_2_ and 15d-PGJ_2_ in cell-free culture media was performed according to previous studies [30,31]. To detect PGD_2_ and 15d-PGJ_2_ in the culture medium, freshly prepared 15 μM PGD_2_ was made by diluting a stock solution in cell-free DMEM containing 10% FBS and 1% penicillin–streptomycin, which was incubated in a humidified atmosphere containing 5% CO_2_ at 37 °C. The culture medium was collected at 0, 0.5, 1, 2, 4, and 24 h, homogenized in methyl acetate, and centrifuged at 10,000× *g* for 10 min at 4 °C. The supernatant was evaporated, and then the residue was reconstituted in the assay buffer. PGD_2_ was measured using the Prostaglandin D_2_–MOX EIA Kit (Cayman Chemical, Ann Arbor, MI, USA) according to the manufacturer’s protocol. The absorbance of samples was measured using the microplate reader SH-1000Lab (Corona Electric, Ibaraki, Japan) at a test wavelength of 412 nm. Similarly, 15d-PGJ_2_ was measured according to the protocol of the 15-deoxy-Δ^12,14^-PGJ_2_ ELISA kit (Enzo Life Sciences, Farmingdale, NY, USA). The absorbance of samples was measured using SH-1000Lab (Corona Electric, Ibaraki, Japan) at test and reference wavelengths of 405 and 570 nm, respectively.

### 2.6. Live/Dead Assay

The amount of viable and dead cells was measured by LIVE/DEAD^®^ Viability/Cytotoxicity Kit for mammalian cells (Molecular Probes, Eugene, OR, USA) according to a previously described method [27]. The images were collected with an inversed fluorescence microscope (IX70, Olympus, Tokyo, Japan) and cell mortality was determined by calculating the percentage of EthD-1-positive cells of the total number of cells (the sum of calcein-positive live cells and EthD-1-positive dead cells).

### 2.7. Statistical Analyses

Data analyses were performed using GraphPad Prism 6.0 (GraphPad Software, San Diego CA, USA). Data are expressed as mean ± standard error of the mean (SEM) or standard deviation (SD). Statistical significance was assessed by Student’s *t*-test or one-way analysis of variance (ANOVA) followed by post hoc Tukey’s multiple tests. Differences at *p* < 0.05 were considered statistically significant.

## 3. Results

### 3.1. PGD_2_ Affects Neurite Outgrowth

**Figure 1:**
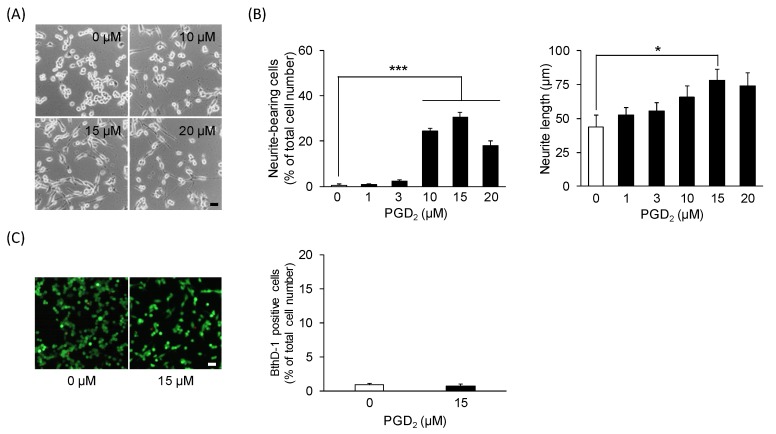
The effect of prostaglandin D_2_ (PGD_2_) on neurite outgrowth. Undifferentiated NSC-34 cells were exposed to various concentrations of PGD_2_ for 24 h. (**A**) Representative phase-contrast microscopy images of NSC-34 cells treated with various concentrations of PGD_2_. Scale bar: 50 μm. (**B**) The percentage of neurite-bearing cells (left panel) and the average neurite length (right panel) in each treatment group. Each value represents the mean ± SEM of four different experiments. * *p* < 0.05, *** *p* < 0.001. (**C**) Photographs show typical fluorescence images double staining of calcein-AM (green, live cells) and EthD-1 (red, dead cells) in each treatment group. Scale bar: 50 μm. Graphs show the percentage of EthD-1-positive dead cells in these cells. Each value represents the mean ± SEM of four different experiments.

First, we investigated the effect of exogenously applied PGD_2_ on neuritogenesis in undifferentiated NSC-34 cells. Neurite-bearing cells were observed in cells treated with ≥10 μM PGD_2_ for 24 h, whereas 0 μM PGD_2_ (vehicle)-treated cells were mostly round in shape (Figure 1A). Moreover, we analyzed the percentage of neurite-bearing cells and the neurite length in each treatment group. Exposure of undifferentiated NSC-34 cells to PGD_2_ (1−20 μM) for 24 h resulted in a significant increase in the percentage of neurite-bearing cells at PGD_2_ concentrations of 10 μM (23.7% ± 1.4%, *p* = 0.0000000506), 15 μM (33.3% ± 2.7%, *p* = 0.00000000114), and 20 μM (20.9% ± 2.1%, *p* = 0.00000543) compared to vehicle-treated cells (0.5% ± 0.3%) (Figure 1B). The maximal effect of PGD_2_ was observed at 15 μM, with an increase of up to approximately 30%. Moreover, the average neurite length was significantly increased in PGD_2_ (15 μM)-treated cells (78.1 ± 7.9 μm, *p* = 0.0218) compared to vehicle-treated cells (43.6 ± 8.7 μm) (Figure 1B). Moreover, we investigated the cytotoxicity of PGD_2_ for undifferentiated NSC-34 cells. As shown in Figure 1C, exposure of differentiated NSC-34 cells to 15 μM PGD_2_ for 24 h did not change the percentage of dead cells stained by EthD-1.

### 3.2. DP1 and DP2 Agonists Do Not Affect Neurite Outgrowth

**Figure 2:**
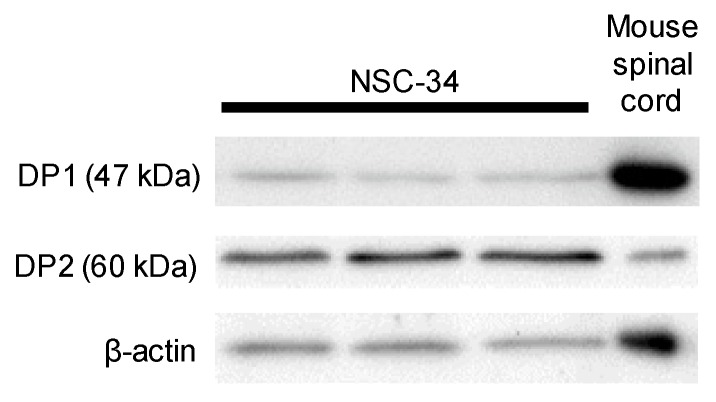
Western blots of D-prostanoid receptor (DP) expression in undifferentiated NSC-34 cells. Mouse spinal cord lysate was used as a positive control. β-actin was used as an internal control. Data from three separate experiments are presented.

Next, we tested whether DP1 and DP2 were expressed in undifferentiated NSC-34 cells by Western blotting. Similar to the reported DP1 and DP2 expression in the mouse spinal cord [23], we detected protein expression of both DP1 and DP2 in undifferentiated NSC-34 cells (Figure 2).

**Figure 3:**
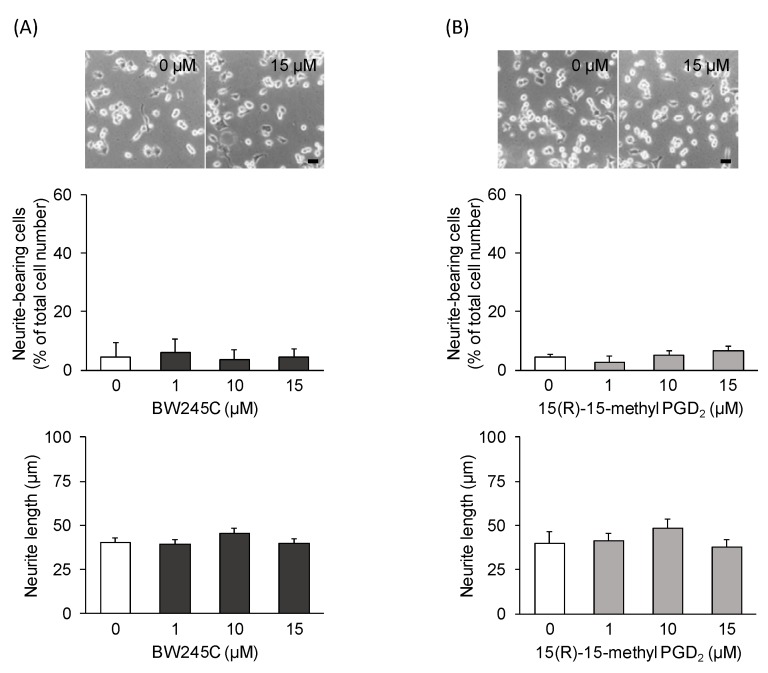
The effects of DP agonists on neurite outgrowth. Undifferentiated NSC-34 cells were exposed to various concentrations of BW245C (a DP1 agonist) or 15(R)-15-methyl PGD_2_ (a DP2 agonist) for 24 h. (**A**) Representative phase-contrast microscopy images of NSC-34 cells treated with various concentrations of BW245C (upper panel). Scale bar: 50 μm. Graphs show the percentage of neurite-bearing cells (middle panel) and the average neurite length (lower panel) in each treatment group. (**B**) Representative phase-contrast microscopy images of NSC-34 cells treated with various concentrations of 15(R)-15-methyl PGD_2_ (upper panel). Scale bar: 50 μm. Graphs show the percentage of neurite-bearing cells (middle panel) and the average neurite length (lower panel) in each treatment group. Each value represents the mean ± SEM of four different experiments.

**Figure 4:**
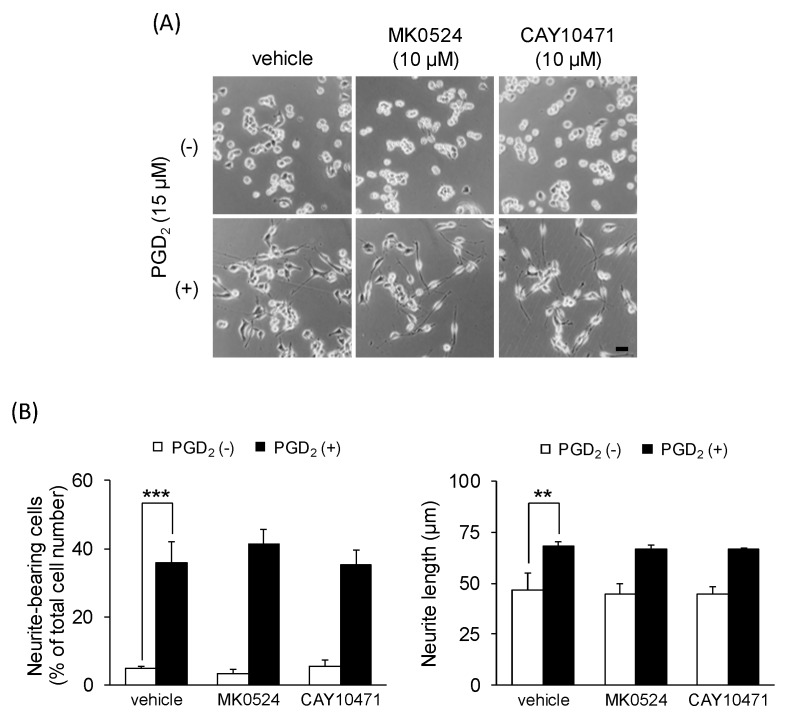
The effects of DP antagonists on PGD_2_-induced neurite outgrowth. Undifferentiated NSC-34 cells were treated with 15 μM PGD_2_ in the presence or absence of 10 μM MK0524 or 10 μM CAY10471 for 24 h. (**A**) Representative phase-contrast microscopy images in each treatment group. Scale bar: 50 μm. (**B**) Graphs show the percentage of neurite-bearing cells (left panel) and the average neurite length (right panel) in each treatment group. Each value represents the mean ± SEM of four different experiments. ** *p* < 0.01, *** *p* < 0.001.

To characterize the subtype(s) of DP responsible for PGD_2_-induced neurite outgrowth in undifferentiated NSC-34 cells, we investigated the effects of two well-characterized DP agonists, BW245C (a DP1 agonist) and 15(R)-15-methyl PGD_2_ (a DP2 agonist). Phase-contrast microscopy revealed no neurite outgrowth in these agonist (15 μM)-treated cells for 24 h (Figure 3A,B). Exposure to BW245C or 15(R)-15-methyl PGD_2_ exerted no significant effect on the percentage of neurite-bearing cells and the average neurite length within the concentration range tested (1−15 μM) (Figure 3A,B). Moreover, co-treatment with MK0524 (a DP1 antagonist, 10 μM) or CAY10471 (a DP2 antagonist, 10 μM) and PGD_2_ (15 μM) had no effect on PGD_2_-induced neurite outgrowth (Figure 4A). There were no significant differences in the percentage of neurite-bearing cells or neurite length between the cells co-treated with MK0524 or CAY10471 and PGD_2_, and the cells treated with PGD_2_ alone (Figure 4B).

### 3.3. 15d-PGJ_2_ Affects Neurite Outgrowth

**Figure 5:**
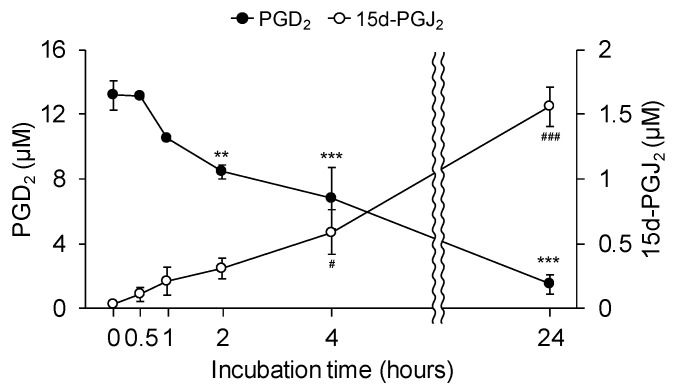
Conversion of PGD_2_ to 15-deoxy-Δ^12,14^-prostaglandin J_2_ (15d-PGJ_2_) under cell-free conditions. Freshly prepared 15 μM PGD_2_ was made by diluting a stock solution in cell-free culture medium. Time-dependent concentrations of PGD_2_ and 15d-PGJ_2_ were measured at 0, 0.5, 1, 2, 4, and 24 h. The left and right axes represent the concentration of PGD_2_ (black circles) and 15d-PGJ_2_ (white circles), respectively. Each value represents the mean ± SEM of three different experiments. ** *p* < 0.01, *** *p* < 0.001 vs. PGD_2_ at 0 h. ^#^ *p* < 0.05, ^###^ *p* < 0.001 vs. 15d-PGJ_2_ at 0 h.

**Figure 6:**
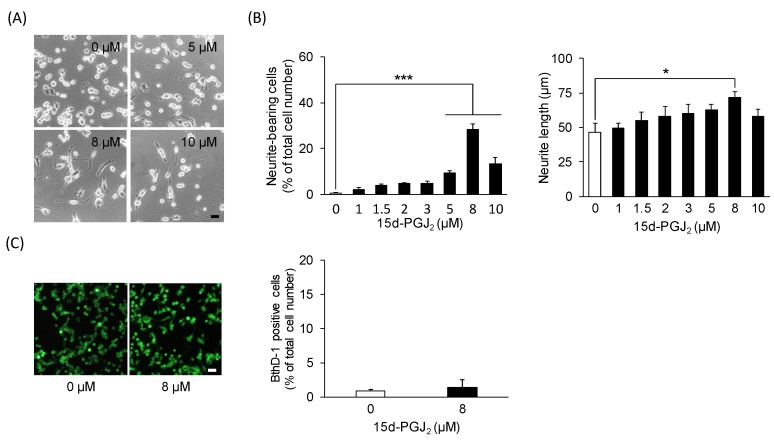
The effect of 15d-PGJ_2_ on neurite outgrowth. (**A**) Representative phase-contrast microscopy images of NSC-34 cells treated with various concentrations of 15d-PGJ_2_. Scale bar: 50 μm. (**B**) Graphs show the percentage of neurite-bearing cells (left panel) and the average neurite length (right panel) in each treatment group. Each value represents the mean ± SEM of four different experiments. * *p* < 0.05, *** *p* < 0.001. (**C**) Photographs show typical fluorescence images of calcein-AM (green, live cells) and EthD-1 (red, dead cells) double staining in each treatment group. Scale bar: 50 μm. Graphs show the percentage of EthD-1-positive dead cells. Each value represents the mean ± SEM of four different experiments.

PGD_2_ is unstable and readily undergoes dehydration to yield the PPARγ natural ligand 15d-PGJ_2_ [32]. Therefore, we sought to identify the conversion of PGD_2_ to 15d-PGJ_2_ in cell-free media. PGD_2_ (15 μM) was incubated under cell-free conditions, and the concentrations of PGD_2_ and 15d-PGJ_2_ were examined by ELISA. The initial concentration of PGD_2_ was 13.2 ± 0.9 μM at 0 h and significantly decreased in a time-dependent manner after incubation for 2 h (8.4 ± 0.4 μM, *p* = 0.00751), 4 h (6.8 ± 1.9 μM, *p* = 0.000640), and 24 h (1.5 ± 0.6 μM, *p* = 0.00000146) (Figure 5). In contrast, the concentration of 15d-PGJ_2_ significantly increased in a time-dependent manner from 32.2 ± 2.1 nM at 0 h to 0.6 ± 0.2 μM (*p* = 0.0103) after 4 h and 1.6 ± 0.2 μM (*p* = 0.000000717) after 24 h (Figure 5). Next, we investigated the effect of exogenously applied 15d-PGJ_2_ on neurite outgrowth in undifferentiated NSC-34 cells. Neurite-bearing cells were observed after 24 h with 15d-PGJ_2_ concentrations of ≥5 μM (Figure 6A). Significantly higher increases in the percentage of neurite-bearing cells were observed at concentrations of 5 μM (9.4% ± 0.8%, *p* = 0.000913), 8 μM (28.4% ± 2.5%, *p* = 0.000000000000843), and 10 μM (13.3% ± 2.7%, *p* = 0.00000414), than in vehicle-treated cells (0.7% ± 0.2%) (Figure 6B). Moreover, 15d-PGJ_2_ (8 μM)-treated cells exhibited significantly longer neurites (71.6 ± 4.4 μm, *p* = 0.0199) than vehicle-treated cells (46.5 ± 6.3 μm) (Figure 6B). Consistent with results from PGD2-treated cells, exposure to 8 μM 15d-PGJ_2_ for 24 h had no significant effect on the percentage of dead cells stained by EthD-1 (Figure 6C).

### 3.4. The PPARγ Antagonist Affects PGD_2_-Induced Neurite Outgrowth

**Figure 7:**
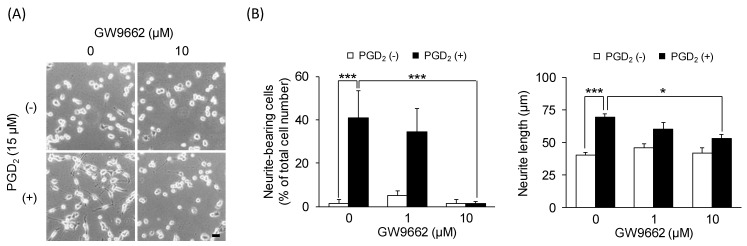
The effect of the peroxisome proliferator-activated receptor γ (PPARγ) antagonist GW9662 on PGD_2_-induced neurite outgrowth. Undifferentiated NSC-34 cells were treated with 15 μM PGD_2_ in the presence or absence of various concentrations of GW9662 for 24 h. (**A**) Representative phase-contrast microscopy images in each treatment group. Scale bar: 50 μm. (**B**) Graphs show the percentage of neurite-bearing cells (left panel) and the average neurite length (right panel) in each treatment group. Each value represents the mean ± SEM of four different experiments. * *p* < 0.05, *** *p* < 0.001.

After confirming the conversion of PGD_2_ to 15d-PGJ_2_ and the effects of 15d-PGJ_2_ on neurite outgrowth, we examined whether treatment with GW9662 (a PPARγ antagonist) prevents PGD_2_-induced neurite outgrowth. The combination of GW9662 (10 μM) and PGD_2_ (15 μM) eliminated almost all the neurite outgrowth observed with PGD_2_ (15 μM) treatment alone (Figure 7A). The morphological change was not observed in the cells treated with GW9662 (10 μM) alone. Neurite outgrowth assays showed that simultaneous treatment with GW9662 (10 μM) and PGD_2_ (15 μM) for 24 h significantly suppressed the PGD_2_ (15 μM)-induced increase in the percentage of neurite-bearing cells from 38.5% ± 6.3% to 2.5% ± 0.8% (*p* = 0.000000542) (Figure 7B (left panel)) and resulted in significantly shorter neurites (53.3 ± 3.0 μm, *p* = 0.0116) than the cells treated with PGD_2_ (15 μM) alone (69.4 ± 2.5 μm) (Figure 7B (right panel)). Moreover, GW9662 (1 μM) tended to suppress the PGD_2_ (15 μM)-induced increase in the percentage of neurite-bearing cells (34.0% ± 0.8%, *p* = 0.872) and elongation of neurites (60.5 ± 4.8 μm, *p* = 0.251) (Figure 7B (left panel)). Treatment with GW9662 (1 and 10 μM) alone had no significant effect on the percentage of neurite-bearing cells and the average neurite length (Figure 7B).

### 3.5. PGD_2_ and 15d-PGJ_2_ Increase the Expression of Motor Neuron-Specific Protein Marker Islet-1

**Figure 8:**
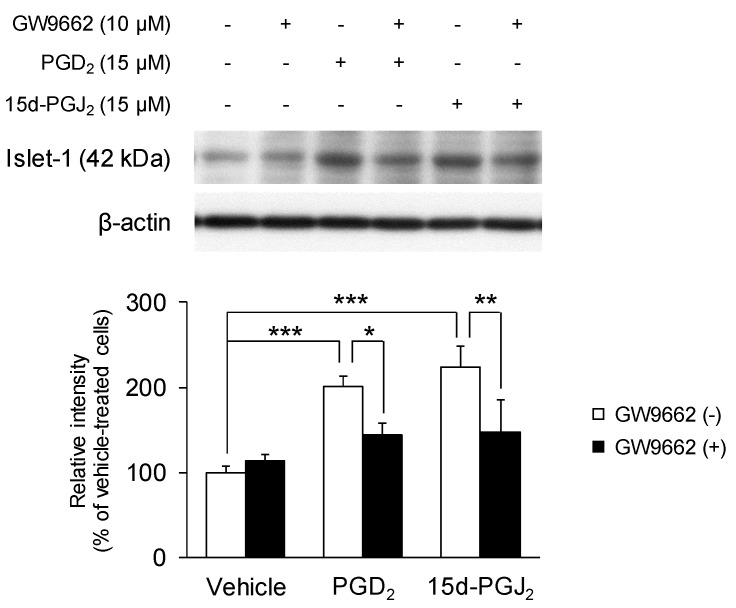
The effects of PGD_2_ and 15d-PGJ_2_ on the expression of the motor neuron-specific protein marker Islet-1. Undifferentiated NSC-34 cells were treated with vehicle (0.04% DMSO), 15 μM PGD_2_, or 8 μM 15d-PGJ_2_ with or without 10 μM GW9662 (a PPARγ antagonist) for 24 h. Equal amounts of cell lysate (10 μg) were analyzed by Western blotting and the Islet-1 antibody and β-actin antibody as an internal control (upper panel). The relative band density was estimated quantitatively using Scion imaging software and is expressed as the ratio of the Islet-1 band intensity relative to the β-actin band intensity. Each value represents the mean ± SD of three different experiments. * *p* < 0.05, ** *p* < 0.01, *** *p* < 0.001.

Lastly, we examined the effects of PGD_2_ and 15d-PGJ_2_ on the expression of the motor neuron-specific protein marker Islet-1 using Western blotting (Figure 8). Undifferentiated NSC-34 cells were treated with vehicle, 15 μM PGD_2_, or 8 μM 15d-PGJ_2_ with or without 10 μM GW9662 for 24 h. The levels of Islet-1, calculated assuming a 100% intensity of Islet-1 expression in vehicle-treated cells, were significantly increased in PGD_2_-treated cells (201.1% ± 12.3%, *p* = 0.000598) and 15d-PGJ_2_ (8 μM)-treated cells (223.7% ± 24.9%, *p* = 0.0000887). The expression of Islet-1 in the cells treated with GW9662 alone remained unchanged (113.0% ± 7.7%, *p* = 0.965). Simultaneous treatment with GW9662 for 24 h significantly suppressed the increased Islet-1 expression caused by PGD_2_ (144.7% ± 13.3%, *p* = 0.0468) and 15d-PGJ_2_ (148.1% ± 37.6%, *p* = 0.00676). 

## 4. Discussion

PGE_2_ plays a role in the differentiation of various cell types, such as regulatory T cells [33], osteoblasts [34], and neurons [35]. Our previous study reported that PGE_2_, which is increased in the spinal cord of ALS patients [36] and model mice [30], induced morphological differentiation of undifferentiated NSC-34 cells [16]. Lipocalin-type PGDS is a major brain-derived protein abundant in human cerebrospinal fluid, second only to albumin [37]. Although PGD_2_ may be present in the CNS in amounts equal to or greater than PGE_2_, the influence of PGD_2_ in motor neuron differentiation remains unclear. In this study, we demonstrated that exposure of undifferentiated NSC-34 cells to PGD_2_ not only increased the percentage of neurite-bearing cells but also elongated neurite length without affecting the number of EthD-1 cells PI-positive dead cells. This suggests that PGD_2_, similar to PGE_2_, induces neuritogenesis in undifferentiated NSC-34 cells without affecting the cell viability.

A previous study reported that DP1 and DP2 are expressed in motor neurons of the adult mouse spinal cord [23]. Consistent with this report, our study demonstrated that DP1 and DP2 are expressed in undifferentiated NSC-34 cells. These results suggest that the characteristics of the model cell used in this study are comparable to those of the motor neurons in mouse spinal cords. Therefore, we investigated whether DP1 and/or DP2 contribute to the effect of PGD_2_ on neurite outgrowth using subtype-specific agonists, BW245C (a DP1 agonist) and 15(R)-15-methyl PGD_2_ (a DP2 agonist). Neither of these two agonists affected neurite outgrowth. Moreover, we showed that a DP1-selective antagonist (MK0524) and a DP2-selective antagonist (CAY10471) were unable to suppress PGD_2_-induced neuritogenesis. These results suggest that PGD_2_-induced neuritogenesis is not mediated by DP1 or DP2 in NSC-34 cells. Our previous study reported that PGE_2_ induced neurite outgrowth by activating EP2 that is coupled to Gs protein [16]. However, our present study indicated that the activation of DP1 did not induce neuritogenesis despite DP1 being the same Gs protein-coupled receptor as EP2. A previous study demonstrated that PGD_2_ (1 μM) caused approximately 25% of DP1 expressed at the membrane level to be internalized in HEK293 cells, reaching a plateau after 120−240 min [38]. However, PGE_2_ (1 μM) caused approximately 40% of EP4 to be rapidly internalized, whereas EP2 was not internalized after 60 min in HEK293 cells [39]. These results imply that one possible explanation for the difference between the effect of DP1 and EP2 on neuritogenesis is that DP1 is internalized and desensitized, whereas EP2 is not.

PGD_2_ is unstable and is spontaneously metabolized to J_2_ prostaglandins including PGJ_2_, Δ^12^-PGJ_2_, and 15d-PGJ_2_ [32]. It has been reported that PGD_2_ is converted to J_2_ prostaglandins in culture medium in the presence of mouse primary cortical neurons, and that a similar pattern of PGD_2_ metabolism is observed in the absence of neurons [40]. In particular, 15d-PGJ_2_ plays an important role in neurite outgrowth of the human neuroblastoma cell line LA–N–5 [41] and rat embryonic midbrain cells [25]. We demonstrated the conversion of PGD_2_ to 15d-PGJ_2_ in culture medium in the absence of NSC-34 cells. Moreover, exposure of NSC-34 cells to 15d-PGJ_2_ resulted in significant neurite outgrowth without affecting the number of EthD-1 cells PI-positive dead cells. These results suggest that the conversion of PGD_2_ to 15d-PGJ_2_ induces neuritogenesis, and also show that PGD_2_ and 15d-PGJ_2_ do not affect the cell survival at the concentration used as a neuritogenesis inducer.

The concentration of PGD_2_ in the CNS is approximately 500 pmol/g tissue (500 pM) in rat hippocampus, 250 pmol/g tissue (250 pM) in rat cerebral cortex [42], and 40 pg/mg tissue (113 pM) in mouse spinal cord [43]. Although the concentration of 15d-PGJ_2_ in the spinal cord remains unclear, the concentration of 15d-PGJ_2_ in the hippocampus was demonstrated to be approximately 8 pmol/g tissue (8 pM) in juvenile rats [42]. These in vivo studies suggest that the concentrations of PGD_2_ and 15d-PGJ_2_ found in the CNS and spinal cord are in the pM range. However, our findings indicated that the effective concentrations of PGD_2_ and 15d-PGJ_2_ on neurite outgrowth in NSC-34 cells were in the μM range. Although the effective concentration of PGD_2_ on neurite outgrowth has not been reported in other in vitro models, 15d-PGJ_2_ has been reported to induce neurite outgrowth in rat embryonic midbrain cells at concentrations of ≥0.5 μM [25]. These results suggest that the necessary concentrations of PGD_2_ and 15d-PGJ_2_ in in vitro studies are higher than physiological concentrations in vivo. In addition, the measured concentrations in in vivo studies represent average tissue concentrations. Considering that prostaglandins act as autocrine or paracrine factors for their target cells, local cellular concentration is more important than average tissue concentration. However, to the best of our knowledge, the local cellular concentrations of PGD_2_ and 15d-PGJ_2_ in the spinal cord are unknown, and thus further studies are needed. Moreover, it is also important to understand how exogenously applied 15d-PGJ_2_ was transported into the cells. Although a previous study reported that exogenously applied 15d-PGJ_2_ was transported into the cells and accumulated in the nucleus [44], the underlying mechanism remains unknown. One possibility is that intracellular uptake was mediated by prostaglandin transporters, similar to that observed with other prostaglandins [43]. Further studies are needed to determine the underlying mechanism of transportation in neurons and its model cells.

15d-PGJ_2_ binds to DP2 with an affinity similar to PGD_2_ [45]. Furthermore, 15d-PGJ_2_ enhancement of nerve growth factor-induced neurite outgrowth is inhibited by CAY10471 (a DP2 antagonist) in PC12 cells [46]. In this study, 15(R)-15-methyl PGD_2_ (a DP2 agonist) did not induce neurite outgrowth, suggesting that DP2 activation is not involved in 15d-PGJ_2_-induced neuritogenesis in NSC-34 cells. Moreover, 15d-PGJ_2_ is commonly known as an endogenous ligand of PPARγ [44]. Indeed, 15d-PGJ_2_ induces differentiation of rat embryonic midbrain cells into dopaminergic neuronal cells in a PPARγ-dependent manner [25]. Nevertheless, 15d-PGJ_2_ is also involved in PPARγ-independent pathways via direct covalent binding to proteins other than PPARγ [47]; 15d-PGJ_2_ enhances nerve growth factor-induced neurite outgrowth via covalent binding to activator protein-1 [48] or the transient receptor potential vanilloid 1 [49] in PC12 cells. These studies suggest that 15d-PGJ_2_-induced neuritogenesis and neuronal differentiation involve pathways that are dependent or independent of PPARγ, according to the cell type. We demonstrated that simultaneous treatment with GW9662 (a PPARγ antagonist) and PGD_2_ for 24 h suppressed PGD_2_-induced neuritogenesis, suggesting that 15d-PGJ_2_ converted from PGD_2_ induces neurite outgrowth via a PPARγ-dependent pathway in undifferentiated NSC-34 cells. 15d-PGJ_2_ contains two electrophilic carbons: the 13-carbon position reacts with the sulfur atom of the cysteine residue in PPARγ, whereas the 9-carbon position within the cyclopentenone ring binds to the cysteine residue in other proteins including nuclear factor-κB, IκB kinase, and activator protein-1 [50]. Further studies are needed to determine how 15d-PGJ_2_ targets proteins such as PPARγ.

Islet-1 is a homeodomain transcription factor that is expressed from the early stages of motor neuron differentiation [51]. The expression of Islet-1 is upregulated during the differentiation of human pluripotent stem cells into motor neurons, and Islet-1 depletion by shRNA prevents Islet-1 expression and motor neuron differentiation [52]. Islet-1 is also expressed in retinoic acid-induced differentiated NSC-34 cells [6]. Therefore, Islet-1 expression is a critical regulator of mature motor neuron formation. In this study, we evaluated Islet-1 expression to determine whether PGD_2_ and 15d-PGJ_2_ promote the maturation of NSC-34 cells into motor neurons. We demonstrated that PGD_2_ and 15d-PGJ_2_ significantly upregulate Islet-1 expression, suggesting that PGD_2_ and 15d-PGJ_2_ can potentiate neural conversion of mature motor neurons. Moreover, we observed that GW9662 (a selective PPARγ antagonist) dramatically inhibited the PGD_2_- and 15d-PGJ_2_-induced increase in Islet-1 expression in differentiated NSC-34 cells. Treatment with 15d-PGJ_2_, retinoic acid, and ciglitazone (a selective PPARγ agonist) suppressed Islet-1 gene expression in neural stem cells generated by culturing dissociated brain cells from newborn mice [53]. In contrast, ciglitazone induced Islet-1 gene expression in human brain tumor stem cell cultures [54]. These results suggest that PPARγ regulates Islet-1 expression at the gene level depending on the type of cell. Although the mechanism underlying the upregulation of Islet-1 warrants further investigation, our results indicate that the PGD_2_- and 15d-PGJ_2_-induced increase in Islet-1 expression was mediated by PPARγ activation.

Several studies have shown that the differentiation of NSC-34 cells leads to neurite outgrowth and the expression of motor neuron-specific proteins [5,6]; as such, these cells can be employed as an in vitro experimental model to study motor neuron dysfunction in response to neurotoxins such as staurosporine, thapsigargin, hydrogen peroxide, homocysteine [55], glutamate [56,57], and cerebrospinal fluid from sporadic ALS patients [58]. However, as pointed out by a previous study [59], the usefulness of differentiated NSC-34 cells has been questioned as these cells exhibit less cell death and a small unsustained increase in the concentration of intracellular calcium after exposure to glutamate compared to primary motor neurons. Our previous study has demonstrated that EP2 and EP3, but not EP1 and EP4, are dominantly expressed in differentiated NSC-34 cells as well as motor neurons in the mouse spinal cord [60]. Similar to the increase in EP2 expression in PGE_2_-treated differentiated NSC-34 cells, EP2 upregulation in motor neurons of ALS model mice was observed at the early symptomatic age [26]. Therefore, these cells are believed to be a suitable model for assessing the response to prostaglandins in motor neurons.

**Figure 9:**
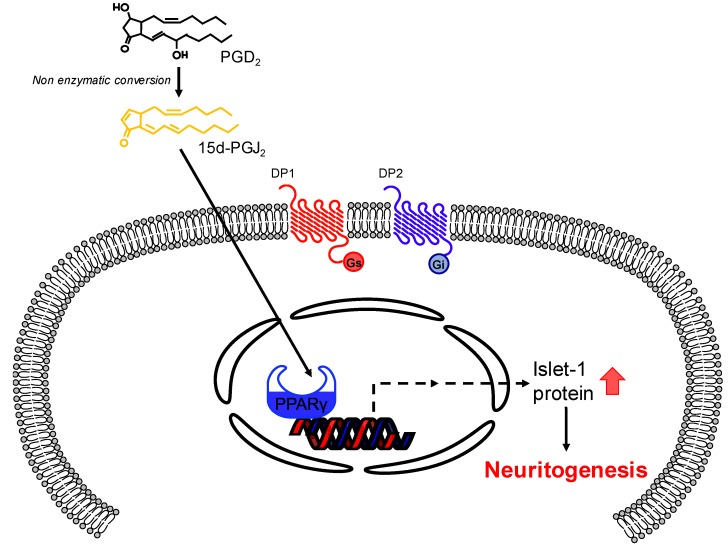
A proposed mechanism underlying PGD_2_-induced neuritogenesis in motor neuron-like NSC-34 cells.

In conclusion, we demonstrated for the first time that exogenously applied PGD_2_ induces neuritogenesis in NSC-34 cells. We suggest that the underlying mechanism involves nonenzymatic conversion of PGD_2_ into 15d-PGJ_2_, which then activates PPARγ (Figure 9). As such, both PGD_2_ and 15d-PGJ_2_ (converted from PGD_2_) promote the differentiation of progenitor cells into motor neurons and could thus be useful for establishing a motor neuron model. This novel effect of PGD_2_ on the differentiation of motor neuron-like cells may provide a potential target for regenerative therapies for motor neuron diseases. 

## References

[1] Arimura N., Kaibuchi K. (2007). Neuronal polarity: From extracellular signals to intracellular mechanisms. Nat. Rev. Neurosci..

[2] Stifani N. (2014). Motor neurons and the generation of spinal motor neuron diversity. Front. Cell. Neurosci..

[3] Jaiswal M.K. (2017). Therapeutic opportunities and challenges of induced pluripotent stem cells-derived motor neurons for treatment of amyotrophic lateral sclerosis and motor neuron disease. Neural Regen. Res..

[4] Cashman N.R., Durham H.D., Blusztajn J.K., Oda K., Tabira T., Shaw I.T., Dahrouge S., Antel J.P., Durham H.D., Tabira T. (1992). Neuroblastoma × spinal cord (NSC) hybrid cell lines resemble developing motor neurons. Dev. Dyn..

[5] Maier O., Böhm J., Dahm M., Brück S., Beyer C., Johann S., Böhm J., Beyer C., Dahm M., Brück S. (2013). Differentiated NSC-34 motoneuron-like cells as experimental model for cholinergic neurodegeneration. Neurochem. Int..

[6] Johann S., Dahm M., Kipp M., Zahn U., Beyer C. (2011). Regulation of choline acetyltransferase expression by 17 β-oestradiol in NSC-34 cells and in the spinal cord. J. Neuroendocrinol..

[7] Keilhoff G., Mbou R.P., Lucas B., Schild L. (2019). The differentiation of spinal cord motor neurons is associated with changes of the mitochondrial phospholipid cardiolipin. Neuroscience.

[8] Keilhoff G., Lucas B., Pinkernelle J., Steiner M., Fansa H. (2014). Effects of cerebrolysin on motor-neuron-like NSC-34 cells. Exp. Cell Res..

[9] Kupershmidt L., Weinreb O., Amit T., Mandel S., Carri M.T., Youdim M.B.H. (2009). Neuroprotective and neuritogenic activities of novel multimodal iron-chelating drugs in motor-neuron-like NSC-34 cells and transgenic mouse model of amyotrophic lateral sclerosis. FASEB J..

[10] Yagi H., Ohkawara B., Nakashima H., Ito K., Tsushima M., Ishii H., Noto K., Ohta K., Masuda A., Imagama S. (2015). Zonisamide enhances neurite elongation of primary motor neurons and facilitates peripheral nerve regeneration in vitro and in a mouse model. PLoS ONE.

[11] Phillis J.W., Horrocks L.A., Farooqui A.A. (2006). Cyclooxygenases, lipoxygenases, and epoxygenases in CNS: Their role and involvement in neurological disorders. Brain Res. Rev..

[12] Yagami T., Koma H., Yamamoto Y. (2016). Pathophysiological roles of cyclooxygenases and prostaglandins in the central nervous system. Mol. Neurobiol..

[13] Sugimoto Y., Narumiya S. (2007). Prostaglandin E receptors. J. Biol. Chem..

[14] Hiruma H., Ichikawa T., Kobayashi H., Hoka S., Takenaka T., Kawakami T. (2000). Prostaglandin E2 enhances axonal transport and neuritogenesis in cultured mouse dorsal root ganglion neurons. Neuroscience.

[15] Mitani K., Sekiguchi F., Maeda T., Tanaka Y., Yoshida S., Kawabata A. (2016). The prostaglandin E2/EP4 receptor/cyclic AMP/T-type Ca2+ channel pathway mediates neuritogenesis in sensory neuron-like ND7/23 cells. J. Pharmacol. Sci..

[16] Nango H., Kosuge Y., Miyagishi H., Sugawa K., Ito Y., Ishige K. (2017). Prostaglandin E2 facilitates neurite outgrowth in a motor neuron-like cell line, NSC-34. J. Pharmacol. Sci..

[17] Urade Y., Eguchi N. (2002). Lipocalin-type and hematopoietic prostaglandin D synthases as a novel example of functional convergence. Prostaglandins Other Lipid Mediat..

[18] Urade Y., Kitahama K., Ohishi H., Kaneko T., Mizuno N., Hayaishi O. (1993). Dominant expression of mRNA for prostaglandin D synthase in leptomeninges, choroid plexus, and oligodendrocytes of the adult rat brain. Proc. Natl. Acad. Sci. USA.

[19] Mohri I., Eguchi N., Suzuki K., Urade Y., Taniike M. (2003). Hematopoietic prostaglandin D synthase is expressed in microglia in the developing postnatal mouse brain. Glia.

[20] Liang X., Wu L., Hand T., Andreasson K. (2005). Prostaglandin D2 mediates neuronal protection via the DP1 receptor. J. Neurochem..

[21] Ogorochi T., Narumiya S., Mizuno N., Yamashita K., Miyazaki H., Hayaishi O. (1984). Regional distribution of prostaglandins D2, E2, and F2 alpha and related enzymes in postmortem human brain. J. Neurochem..

[22] Kihara Y., Matsushita T., Kita Y., Uematsu S., Akira S., Kira J.-I., Ishii S., Shimizu T. (2009). Targeted lipidomics reveals mPGES-1-PGE2 as a therapeutic target for multiple sclerosis. Proc. Natl. Acad. Sci. USA.

[23] Grill M., Heinemann A., Hoefler G., Peskar B.A., Schuligoi R. (2008). Effect of endotoxin treatment on the expression and localization of spinal cyclooxygenase, prostaglandin synthases, and PGD2 receptors. J. Neurochem..

[24] Kliewer S.A., Lenhard J.M., Willson T.M., Patel I., Morris D.C., Lehmann J.M. (1995). A prostaglandin J2 metabolite binds peroxisome proliferator-activated receptor γ and promotes adipocyte differentiation. Cell.

[25] Park K.S., Da Lee R., Kang S.-K., Han S.Y., Park K.L., Yang K.H., Song Y.S., Park H.J., Lee Y.M., Yun Y.P. (2004). Neuronal differentiation of embryonic midbrain cells by upregulation of peroxisome proliferator-activated receptor-gamma via the JNK-dependent pathway. Exp. Cell Res..

[26] Kosuge Y., Miyagishi H., Yoneoka Y., Yoneda K., Nango H., Ishige K., Ito Y. (2017). Pathophysiological role of prostaglandin E2-induced up-regulation of the EP2 receptor in motor neuron-like NSC-34 cells and lumbar motor neurons in ALS model mice. Neurochem. Int..

[27] Kosuge Y., Nango H., Kasai H., Yanagi T., Mawatari T., Nishiyama K., Miyagishi H., Ishige K., Ito Y. (2020). Generation of Cellular Reactive Oxygen Species by Activation of the EP2 Receptor Contributes to Prostaglandin E2-Induced Cytotoxicity in Motor Neuron-Like NSC-34 Cells. Oxid. Med. Cell. Longev..

[28] Schneider C.A., Rasband W.S., Eliceiri K.W. (2012). NIH Image to ImageJ: 25 years of image analysis. Nat. Methods.

[29] Meijering E., Jacob M., Sarria J.-C.F., Steiner P., Hirling H., Unser M. (2004). Design and validation of a tool for neurite tracing and analysis in fluorescence microscopy images. Cytometry.

[30] Miyagishi H., Kosuge Y., Takano A., Endo M., Nango H., Yamagata-Murayama S., Hirose D., Kano R., Tanaka Y., Ishige K. (2017). Increased expression of 15-hydroxyprostaglandin dehydrogenase in spinal astrocytes during disease progression in a model of amyotrophic lateral sclerosis. Cell Mol. Neurobiol..

[31] Shibata T., Kondo M., Osawa T., Shibata N., Kobayashi M., Uchida K. (2002). 15-Deoxy-Δ 12,14 -prostaglandin J 2. J. Biol. Chem..

[32] Straus D.S., Glass C.K. (2001). Cyclopentenone prostaglandins: New insights on biological activities and cellular targets. Med. Res. Rev..

[33] Li H., Chen H.-Y., Liu W.-X., Jia X.-X., Zhang J.-G., Ma C.-L., Zhang X.-J., Yu F., Cong B. (2017). Prostaglandin E2 restrains human Treg cell differentiation via E prostanoid receptor 2-protein kinase A signaling. Immunol. Lett..

[34] Liu J., Chen L., Tao X., Tang K. (2013). Phosphoinositide 3-kinase/Akt signaling is essential for prostaglandin E2-induced osteogenic differentiation of rat tendon stem cells. Biochem. Biophys. Res. Commun..

[35] Wong C.T., Ussyshkin N., Ahmad E., Rai-Bhogal R., Li H., Crawford D.A. (2016). Prostaglandin E2 promotes neural proliferation and differentiation and regulates Wnt target gene expression. J. Neurosci. Res..

[36] Almer G., Teismann P., Stevic Z., Halaschek-Wiener J., Deecke L., Kostic V., Przedborski S., Halaschek–Wiener J., Deecke L., Kostic V. (2002). Increased levels of the pro-inflammatory prostaglandin PGE2 in CSF from ALS patients. Neurology.

[37] Reiber H. (2001). Dynamics of brain-derived proteins in cerebrospinal fluid. Clin. Chim. Acta.

[38] Gallant M.A., Slipetz D., Hamelin É., Rochdi M.D., Talbot S., De Brum-Fernandes A.J., Parent J.L. (2007). Differential regulation of the signaling and trafficking of the two prostaglandin D2 receptors, prostanoid DP receptor and CRTH2. Eur. J. Pharmacol..

[39] Desai S., April H., Nwaneshiudu C., Ashby B. (2000). Comparison of agonist-induced internalization of the Human EP2 and EP4 Prostaglandin Receptors: Role of the Carboxyl Terminus in EP4 Receptor Sequestration. Mol. Pharmacol..

[40] Liu H., Li W., Rose M.E., Pascoe J.L., Miller T.M., Ahmad M., Poloyac S.M., Hickey R.W., Graham S.H. (2013). Prostaglandin D2 toxicity in primary neurons is mediated through its bioactive cyclopentenone metabolites. Neurotoxicology.

[41] Han S.W., Greene M.E., Pitts J., Wada R.K., Sidell N. (2001). Novel expression and function of peroxisome proliferator-activated receptor gamma (PPARϒ) in human neuroblastoma cells. Clin. Cancer Res..

[42] Liu H., Rose M.E., Miller T.M., Li W., Shinde S.N., Pickrell A.M., Poloyac S.M., Graham S.H., Hickey R.W. (2013). COX2-derived primary and cyclopentenone prostaglandins are increased after asphyxial cardiac arrest. Brain Res..

[43] Figueiredo-Pereira M.E., Rockwell P., Schmidt-Glenewinkel T., Serrano P. (2015). Neuroinflammation and J2 prostaglandins: Linking impairment of the ubiquitin-proteasome pathway and mitochondria to neurodegeneration. Front. Mol. Neurosci..

[44] Hilliard M., Frohnert C., Spillner C., Marcone S., Nath A., Lampe T., Fitzgerald D.J., Kehlenbach R.H. (2010). The anti-inflammatory prostaglandin 15-deoxy-delta(12,14)-PGJ2 inhibits CRM1-dependent nuclear protein export. J. Biol. Chem..

[45] Sawyer N., Cauchon E., Chateauneuf A., Cruz R.P.G., Nicholson D.W., Metters K.M., O’Neill G.P., Gervais F.G. (2002). Molecular pharmacology of the human prostaglandin D2 receptor, CRTH2. Br. J. Pharmacol..

[46] Hatanaka M., Shibata N., Shintani N., Haba R., Hayata A., Hashimoto H., Baba A. (2010). 15d-prostaglandin J2 enhancement of nerve growth factor–induced neurite outgrowth is blocked by the chemoattractant receptor– homologous molecule expressed on T-Helper Type 2 Cells (CRTH2) antagonist CAY10471 in PC12 Cells. J. Pharmacol. Sci..

[47] Shibata T. (2015). 15-Deoxy-Δ^12^,^14^-prostaglandin J₂ as an electrophilic mediator. Biosci. Biotechnol. Biochem..

[48] Jung K.M., Park K.S., Oh J.H., Jung S.Y., Yang K.H., Song Y.S., Son D.J., Park Y.H., Yun Y.P., Lee M.K. (2003). Activation of p38 mitogen-activated protein kinase and activator Protein-1 during the promotion of neurite extension of PC-12 Cells by 15-deoxy-Δ 12,14 -prostaglandin J2. Mol. Pharmacol..

[49] Shibata T., Takahashi K., Matsubara Y., Inuzuka E., Nakashima F., Takahashi N., Kozai D., Mori Y., Uchida K. (2016). Identification of a prostaglandin D2 metabolite as a neuritogenesis enhancer targeting the TRPV1 ion channel. Sci. Rep..

[50] Shiraki T., Kamiya N., Shiki S., Kodama T.S., Kakizuka A., Jingami H. (2005). α,β-Unsaturated ketone is a core moiety of natural ligands for covalent binding to peroxisome proliferator-activated receptor γ. J. Biol. Chem..

[51] Ericson J., Thor S., Edlund T., Jessell T.M., Yamada T. (1992). Early stages of motor neuron differentiation revealed by expression of homeobox gene Islet-1. Science.

[52] Qu Q., Li D., Louis K.R., Li X., Yang H., Sun Q., Crandall S.R., Tsang S., Zhou J., Cox C.L. (2014). High-efficiency motor neuron differentiation from human pluripotent stem cells and the function of Islet-1. Nat. Commun..

[53] Kanakasabai S., Pestereva E., Chearwae W., Gupta S.K., Ansari S., Bright J.J. (2012). PPARγ agonists promote oligodendrocyte differentiation of neural stem cells by modulating stemness and differentiation genes. PLoS ONE.

[54] Pestereva E., Kanakasabai S., Bright J.J. (2012). PPARγ agonists regulate the expression of stemness and differentiation genes in brain tumour stem cells. Br. J. Cancer.

[55] Hemendinger R.A., Armstrong E.J., Radio N., Brooks B.R. (2012). Neurotoxic injury pathways in differentiated mouse motor neuron-neuroblastoma hybrid (NSC-34D) cells in vitro-limited effect of riluzole on thapsigargin, but not staurosporine, hydrogen peroxide and homocysteine neurotoxicity. Toxicol. Appl. Pharmacol..

[56] Liu X., Xu S., Wang P., Wang W. (2015). Transient mitochondrial permeability transition mediates excitotoxicity in glutamate-sensitive NSC34D motor neuron-like cells. Exp. Neurol..

[57] Eggett C.J., Crosier S., Manning P., Cookson M.R., Menzies F.M., McNeil C.J., Shaw P.J. (2000). Development and characterisation of a glutamate-sensitive motor neurone cell line. J. Neurochem..

[58] Vijayalakshmi K., Ostwal P., Sumitha R., Shruthi S., Varghese A.M., Mishra P., Manohari S.G., Sagar B.C., Sathyaprabha T.N., Nalini A. (2015). Role of VEGF and VEGFR2 receptor in reversal of ALS-CSF induced degeneration of NSC-34 motor neuron cell line. Mol. Neurobiol..

[59] Madji Hounoum B., Vourc’h P., Felix R., Corcia P., Patin F., Guéguinou M., Potier-Cartereau M., Vandier C., Raoul C., Andres C.R. (2016). NSC-34 motor neuron-like cells are unsuitable as experimental model for glutamate-mediated excitotoxicity. Front. Cell. Neurosci..

[60] Miyagishi H., Kosuge Y., Yoneoka Y., Ozone M., Endo M., Osada N., Ishige K., Kusama-Eguchi K., Ito Y. (2013). Prostaglandin E2-induced cell death is mediated by activation of EP2 receptors in motor neuron-like NSC-34 cells. J. Pharmacol. Sci..

